# Developing a model for analysis the extra costs associated with surgical site infections (SSIs): an orthopaedic and traumatological study run by the Gaetano Pini Orthopaedic Institute

**DOI:** 10.1186/2047-2994-4-S1-P68

**Published:** 2015-06-16

**Authors:** M Nobile, P Navone, A Orzella, R Colciago, F Auxilia, G Calori

**Affiliations:** 1 Orthopedic Institute, G. Pini, Milan, Italy; 2 Phd Program in Public Health, University of Milan, Milan, Italy; 3 Aon Hewitt Risk & Consulting AGRC Italy, Milan, Italy; 4 Department of Biomedical Sciences for Health, University of Milan, Milan, Italy; 5 Orthopedic Institute, G. Pini, Milan, Milan, Italy

## Introduction

Over the years, the Gaetano Pini Orthopaedic Institute has implemented several projects in the hope of preventing healthcare-associated infections (HAIs).

In particular, the institute participated in a long-term project monitoring arthroplasty-associated SSIs in 2010 (the ISChIA study), funded by the Italian Society of Hygiene’s GISIO (the Italian Study Group of Hospital Hygiene) which involved several different hospitals and enabled it to assess the frequency of SSIs in this kind of operation and identify potential risk factors.

## Objectives

This project aims to develop a model for assessing the direct cost of SSIs borne by the hospital and the regional health service. The study was run by the Gaetano Pini Orthopaedic Institute in partnership with Aon Hewitt Risk & Consulting, AGRC Italy and the University of Milan.

## Methods

Seven patients who developed an SSI during the ISChIA project were chosen for the study. A model for analysing the additional costs of an SSI was developed which started by considering two different circumstances: hospital admission with changes to the DRG attributable to the SSI and cases where the extra costs could be traced back to subsequent hospital admissions or outpatient procedures.

Moreover, an analysis aimed at establishing the costs borne by the hospital and those borne by the regional health service was carried out.

## Results

The total extra cost of the SSIs analysed came to over €32,000, with an average cost per SSI of approximately €9,560 and costs ranging from €3,411 to €22,273 for hospital admissions due to infections. The cost of €57,419 was borne by the regional health service – with an average cost per case of €8,202 – while the hospital had to pay €8,513 with an average cost per case of €1,216.

## Conclusion

This project has allowed us to develop a quantitative model for assessing the extra costs associated with SSIs. Certain variables that could lead to an underestimation of the results need to be taken into consideration.

Of these, the lack of post-discharge information and the fragmentary nature of such data could affect the clinical outcome and the possibility of quantifying the true cost.

## Disclosure of interest

None declared.

